# Exercise Training and Biomarkers of Neuroaxonal Injury in Multiple Sclerosis: Narrative Review

**DOI:** 10.3390/ijerph23030380

**Published:** 2026-03-17

**Authors:** Trevor B. Martin, Noah G. Dubose, Robert W. Motl

**Affiliations:** Department of Kinesiology and Nutrition, University of Illinois Chicago, Chicago, IL 60612, USA

**Keywords:** multiple sclerosis, neuroaxonal damage, serum biomarkers, neurofilament light chain protein, glial fibrillary acidic protein, exercise training

## Abstract

**Highlights:**

**Public health relevance—how does this work relate to a public health issue?**

- This review examines how exercise training may influence sNfL and sGFAP levels, as key biomarkers of disease activity and neurodegeneration in MS.

**Public health significance—why is this work of significance to public health?**

- Understanding how biomarker levels may be altered by exercise training could advance the identification and understanding of mechanistic pathways driving disability and inform more targeted approaches for interventions.

**Public health implications—what are the key implications or messages for practitioners, policy makers and/or researchers in public health?**

- The findings could guide the design of future RCTs concerning sNfL and sGFAP as accessible, cost-effective, and measurable indicators of the neuroprotective effects of exercise training.
- Implementation of the discovery and experimental medicine models offers a way to strengthen RCT design and supports evidence-based exercise recommendations in MS.

**Abstract:**

There is increasing interest in exercise training (ET) as a behavior with potential disease-modifying properties in people with multiple sclerosis (MS), as ET has beneficial effects on relapses, lesions, disability, and cognitive-processing speed (CPS) as markers of MS disease progression. There is limited evidence for ET as a behavior that may have disease-modifying properties based on its association with body fluid biomarkers of neuroaxonal injury in MS. This paper involved a narrative review for building a rationale that supports focusing on ET and body fluid biomarkers of neuroaxonal injury in MS, namely, neurofilament light chain (NfL) and glial fibrillary acidic protein (GFAP). We searched the PubMed, EBSCOhost (Medline Ultimate), and EMBASE databases from inception through February 2026 for articles aligning with the focus of this narrative review. The articles indicated that sNfL and sGFAP levels were notably higher in MS than in controls; differed across demographic and clinical variables; and responded beneficially with disease-modifying therapy usage in MS. We further located two meta-analyses examining ET effects on sNfL and sGFAP in MS, and there were significant positive effects on sNfL, but not sGFAP. Researchers should adopt discovery models and experimental medicine frameworks for guiding future randomized controlled trials of ET and biomarkers of neuroaxonal injury in MS.

## 1. Introduction

There are an estimated 1 million adults living with multiple sclerosis (MS) in the United States [1]. MS is an immune-mediated and neurodegenerative disease resulting in demyelination and transection of axons and loss of neurons in the central nervous system (CNS) [2]. The extent and location of CNS damage (e.g., lesions) manifest as neurological disability, cognitive and walking dysfunction, symptoms, and compromised participation and quality of life (QOL).

Disease-modifying therapies (DMTs) are the primary approach for management of MS itself, and those medications are effective for reducing rates of relapses, lesions, and disability progression [3]. Nevertheless, people with MS still demonstrate cognitive and walking dysfunction and symptoms, perhaps based on residual CNS damage and/or smoldering disease as well as alterations in health behaviors resulting in comorbidity [4,5]. Exercise training (ET) has been proposed as a behavior with potential disease-modifying properties in MS, such that benefits extend beyond symptom management and rehabilitation alone [6]. ET is a subtype of physical activity that involves planned, structured, and repetitive movement of the body through skeletal muscle contraction that results in a substantial increase in the rate of energy expenditure over resting values with the intention of maintaining or improving health-related fitness [7]. ET has been associated with beneficial effects on relapse rate, lesion number and volume, disability progression, and cognitive processing speed (CPS) in MS, thereby suggesting it as a supplemental behavioral intervention in MS [6,8]. We further note that two meta-analyses have reported a large, statistically significant effect of ET on brain-derived neurotrophic factor (BDNF) (ES range = 0.78–2.40), yet smaller, non-significant effects on nerve growth factor (NGF) (ES = 0.28) and ciliary neurotrophic factor (CNTF) (ES = 0.24) in MS [9,10].

To date, there is limited but growing interest in exploring ET and its associations with body fluid biomarkers of neuroaxonal injury in the CNS [5,8]. The focus on body fluid biomarkers (i.e., objective, quantifiable biomedical markers) such as serum neurofilament light chain (sNfL) or glial fibrillary acidic protein (sGFAP) is important for positioning ET as a potential modifier of latent neuroaxonal injury (i.e., disease activity) and establishing its biological basis as a behavior with the potential for modifying the disease. There is initial evidence for beneficial effects of ET, reducing sNfL levels in MS (ES = −0.55), from a recent meta-analysis, thereby supporting the provision of a focal paper on this topic for advancing the study of ET [11]. There is further expanding knowledge regarding the potential mechanisms explaining how ET may be associated with downstream adaptation or preservation of CNS integrity (i.e., NfL or GFAP), and the primary candidates include the irisin and kynurenine pathways [12,13,14,15]. Irisin is a myokine released with muscular contraction, and has been implicated in neuroprotective mechanisms, including the expression of neuroprotective factors such as BDNF [13,15,16,17]. The kynurenine pathway's metabolism is activated with muscular contraction. The ratio of key metabolites produced along this pathway has been implicated in the activation or suppression of inflammatory activity in the CNS [12,18,19]. Those pathways may be associated with markers of neuroaxonal injury, such as NfL or GFAP, by promoting expression of neuroprotective molecules like BDNF, or via a shift of the inflammatory profile within the CNS that may lessen the extent of neuroaxonal injury occurring and, consequently, the amount of NfL or GFAP.

This paper involved a narrative review for building a rationale that supports focusing on ET and body fluid biomarkers of neuroaxonal injury in MS, namely, NfL and GFAP. This is important for advancing our understanding of the benefits of ET beyond fitness, function, symptoms, and quality of life toward body fluid biomarkers of neuroaxonal injury in MS. Such a focus may position ET as a health behavior that could be implemented alongside DMTs within the clinical armamentarium of MS management. This narrative review was conducted based on the Scale for the Assessment of Narrative Review Articles (SANRA) guidelines [20] and provides an overview and description of common body fluid biomarkers of neuroaxonal injury (e.g., sNfL and sGFAP) in MS, and then focuses on four topical areas including comparison of sNfL and sGFAP levels between MS and controls; examination of sNfL and GFAP across demographic and clinical variables in MS; documentation of DMT's effects on sNfL and sGFAP; and description of existing research examining ET's effects on sNfL and sGFAP levels in MS. We completed this paper with a discussion of future research avenues involving a discovery model combined with an experimental medicine framework for advancing the study of ET and body fluid biomarkers of neuroaxonal injury in MS [21,22].

### Search Strategy

The search strategy was guided by the SANRA guidelines. We originally searched the PubMed database from inception through August of 2025 for articles relevant to four topical areas: comparison of sNfL and sGFAP levels between MS and controls; examination of sNfL and GFAP across demographic and clinical variables in MS; and documentation of disease-modifying therapy (DMT)'s effects on sNfL and sGFAP. We originally searched only PubMed because it is a premier, comprehensive database covering biomedical and health sciences research topics and is widely considered a reliable and comprehensive database for health-related topics when compared with other databases [23]. We searched PubMed using the terms “serum neurofilament light chain” and “serum glial fibrillary acidic protein” in combination with keywords specific to each section of the narrative review (e.g., “age”, “BMI”, “cognitive function”, “disease-modifying therapies”, and “optical coherence tomography”). The exploration of the above-mentioned sections involved no formal risk-of-bias assessment based on the breadth of the literature per section. The studies included provided the conceptual contributions for our focus on ET and body fluid biomarkers of neuroaxonal injury. Articles located in the initial search were title- and abstract-screened and then included if the article focused on people with MS, assessed the variable of interest for a particular section, and quantified sNfL or sGFAP as outcomes. There was a tremendous breadth of literature and a number of articles per section, and, therefore, we focused on individual articles (both original and meta-analytic) that would best support the scope of this manuscript.

**Figure 1:**
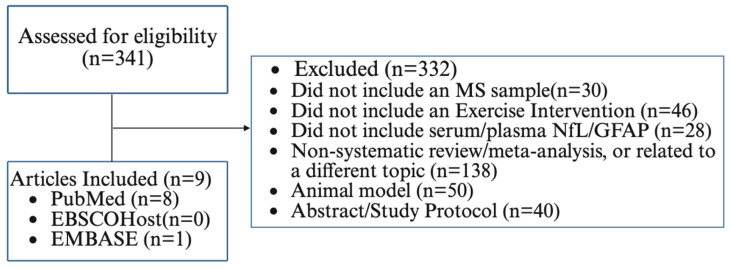
Flow Diagram for Literature Screening and Study Inclusion.

We repeated a separate search specifically for studies involving sNfL and sGFAP along with ET using the PubMed, EBSCOhost (Medline Ultimate), and EMBASE databases from inception through February 2026 (see site-appropriate search strings in Supplemental Document S1). The search identified articles comprising clinical and randomized trials, systematic reviews, and meta-analyses, yielding 48 records from PubMed, 262 from EBSCOhost, and 31 from EMBASE. The 341 records were title- and abstract-screened, and we identified two meta-analyses and one additional article from the updated searches for inclusion in this manuscript. We included detailed information from nine total articles, eight of which were included in two meta-analyses, for granular details on the population, intervention, control, outcomes, and study design. We excluded articles that focused on a neurological population outside of MS; did not include an ET intervention; did not include a serum/plasma measure of NfL or GFAP; involved a topic area outside the scope of this review; included animal models; or were abstracts/study protocols. The results of the search are reported in Figure 1. We further provided a formal risk-of-bias assessment and identified methodological weaknesses of the literature on ET and the biomarkers of neuroaxonal injury.

## 2. Common Body Fluid Biomarkers of Neuroaxonal Injury: Overview and Description

Neuroaxonal injury is a pathophysiological substrate of permanent disability in various neurological diseases, including MS. To that end, neurofilament proteins have emerged as candidate biomarkers of neuroaxonal damage in MS, as neurofilaments are released into the CSF and, ultimately, the blood during neuroaxonal damage and degeneration. The two most common biomarkers of neuroaxonal damage studied in neurological diseases, including MS, are neurofilament light chain (NfL) and glial fibrillary acidic protein (GFAP).

The cytoskeleton of neurons and axons contains five neurofilament protein isoforms, and NfL (IV) is the most abundant of the five. The primary function of NfL is the mechanical stabilization of the axonal cytoskeleton, but other functions include regulation of axonal diameter, flow, and transport. NfL is released from damaged or degenerating axons and neurons into the adjacent interstitial fluid compartments and then diffuses into the surrounding cerebral spinal fluid (CSF) and blood in neurological diseases such as MS [24,25,26]. sNfL concentrations from blood samples correlate with CSF measurements and reflect acute, and perhaps ongoing neuroaxonal damage [27]. This is important as it supports sNfL as an accessible body fluid biomarker of neuroaxonal injury for clinical research and practice.

Glial fibrillary acidic protein (GFAP) is a major intermediate filament (type III) protein primarily expressed by mature astrocytes within the CNS [28]. The primary function of GFAP is to provide structural support for the shape and integrity of astrocytes, as well as regulation of the blood–brain barrier, metabolic support of neurons, controlling local synaptic activity, and influencing the surrounding extracellular environment [29,30]. When astrocytes become active or damaged, GFAP is upregulated and released into the surrounding CNS, eventually diffusing into the CSF and blood in neurological diseases such as MS [30]. This supports sGFAP as another accessible fluid biomarker of neuroaxonal injury involving astrocytes in MS.

### Quantification of Body Fluid Biomarkers of Neuroaxonal Injury

Biomarker detection methods for quantifying MS disease activity include cerebrospinal fluid (CSF), serum, and plasma [31,32,33]. Quantifying NfL and GFAP levels in CSF is considered ideal for measuring neuroaxonal injury because of the biological proximity to the damage occurring and its high analytical performance for quantifying biomarkers. However, it is not a plausible option for regular measurement as the spinal tap process for accessing CSF is highly invasive with a risk factor profile that exceeds its benefits for ET research, and it requires a prolonged period of recovery. To that end, both NfL and GFAP have been routinely measured in blood, either serum or plasma, as a substitute for CSF. There is evidence that the blood-based assessments strongly correlate with CSF values; however, concentrations in blood are significantly lower than in CSF [33,34]. The quantification of NfL and GFAP in serum is most common and is typically conducted using the ELISA and SIMOA assays [35]. Both the ELISA and SIMOA are antibody-based immunoassays that identify targets by generating an enzyme-mediated signal for quantifying biomarker concentrations. ELISA generates a generalized signal based on the cumulative enzymatic activity, whereas SIMOA provides “ultra-sensitive” detection of target analytes by using antibody-coated beads that capture the target. The beads are then filtered onto an array of millions of femtoliter-sized wells, where each well is large enough for one bead. This allows for digital counting of the “on” wells, down to femtomolar concentrations. This extremely low quantification of the analytes using SIMOA far exceeds the sensitivity of ELISA [27]. The preference for blood-based assessment is the SIMOA platform, based on the higher precision offered, but the ELISA approach is reasonable for those who do not have SIMOA technology access. Robust reporting of the approach used in quantification of biomarkers of interest is imperative, specifically within ET studies, as the clear reporting of the platform would improve the comparability and reproducibility of methods used across those trials.

## 3. Body Fluid Biomarkers of Neuroaxonal Injury in MS

### 3.1. Overall Levels of Body Biomarkers Between MS and Controls

There has been substantial research demonstrating that sNfL and sGFAP concentrations are higher in MS than in healthy controls, as would be expected considering MS is an immune-mediated and neurodegenerative disease resulting in demyelination and transection of axons and loss of neurons in the CNS. Indeed, one recent meta-analysis compared sNfL levels between people with MS and controls from 31 published papers [36]. The meta-analysis indicated significantly and moderately higher sNfL levels in MS than in controls (SMD = 0.64; *p* < 0.001) [36]. Another recent meta-analysis compared sGFAP between MS and controls from 49 published papers [37]. sGFAP concentrations were significantly and moderately higher in MS than in controls (SMD = 0.54; *p* < 0.05) [37]. Collectively, those two meta-analyses indicate that sNfL and sGFAP concentrations are higher in MS than in controls, and this seemingly reflects ongoing neuroaxonal injury and damage.

### 3.2. Body Fluid Biomarkers and MS Disease Characteristics

There is increasing evidence for differences in sNfL and sGFAP across MS disease characteristics, and this is important as it might identify specific segments of MS for focal interventions targeting biomarkers of neuroaxonal injury. sNfL concentrations vary by disease type with increasingly higher levels across the different subtypes of MS, including clinically isolated syndrome (CIS), relapsing-remitting MS (RRMS), secondary progressive MS (SPMS), and primary progressive MS (PPMS). Indeed, one meta-analysis indicated higher sNfL in RRMS than in CIS (SMD = 0.30; *p* < 0.001), and in progressive forms of MS (PPMS and SPMS) than in RRMS (SMD = 0.56; *p* < 0.001) [36]. Serum and CSF concentrations of NfL vary depending on disease activity, specifically during a relapse [36,38]. The highest concentrations of sNfL occur during a relapse in RRMS based on ongoing neuroaxonal injury within the CNS. The evidence of differences in sNfL as a function of disease duration is sparse and inconclusive, yet it is generally thought that higher levels of sNfL are present in those with a longer disease duration.

sGFAP concentrations further vary by disease type (i.e., CIS, RRMS, SPMS, and PPMS) [39,40]. For example, sGFAP levels were significantly higher in progressive forms of MS (SPMS and PPMS) than in RRMS (SMD = 0.5; *p* < 0.001), and the higher concentrations of sGFAP in SPMS and PPMS seemingly reflect increased or accrued damage [37]. However, the relationship between disease duration and sGFAP is unclear, and only one study has reported a positive correlation [41].

### 3.3. Body Fluid Biomarkers and Demographic/Morphological Characteristics in MS

There is increasing evidence for variations in sNfL and sGFAP as a function of demographic and morphological characteristics of people with MS, and this is important as these variables might identify confounders and/or unique patient characteristics for examination in focal research (e.g., biological sex, ethnicity, or older age). Chronological age, based on associated cellular senescence, is strongly associated with increases in sNfL and sGFAP in MS [40,42]. Indeed, sNfL concentrations increase by 2.2% annually between the ages of 20 and 50 years and further increase between 50 and 70 years of age in healthy controls [43]. The relationship between age and sNfL in MS remains significant; however, inflammatory activity and MS subtype function as confounders of the relationship [44]. CSF and serum GFAP further increase with age, and the mean annual increases per year differ for controls (6.5 ng/dL), RRMS (8.1 ng/dL), and SPMS (18.9 ng/dL) [40,45]. To date, there have been several studies comparing sNfL and sGFAP by biological sex and race/ethnicity in MS, but the current consensus is that there are not sufficient data for determining definitively if sNfL and sGFAP concentrations differ by sex and race/ethnicity [46,47,48].

Body mass index (BMI) is a morphological factor associated with sNfL and sGFAP in MS, and this might serve as a potential confounder in clinical research. There is evidence that BMI negatively correlates with the central and peripheral measures of NfL and GFAP, and this has been associated with a greater blood volume in persons with MS who have a higher BMI [47,49].

## 4. Clinical Correlates of Body Fluid Biomarkers of Neuroaxonal Injury in MS

### 4.1. MRI Outcomes

There has been focus on the associations between neurofilaments and magnetic resonance imaging (MRI)-derived markers of CNS activity and integrity in MS. For example, higher baseline values of sNfL have been associated with gadolinium enhancing (Gd+) lesions and whole brain volume (β = 0.352; *p* <0.001), white matter volume (β = −0.229; *p* < 0.01), and volumes of the putamen (β = −1.687; *p* =0.012) and thalamus (β = −0.372; *p* < 0.001) in MS [46,50]. Other studies have reported positive correlations between sNfL and lesion volume and number in MS [43,51,52].

There is some research examining sGFAP and its relationship with MRI metrics. For example, higher levels of sGFAP have been associated with MRI metrics such as white matter volume (r = −0.4; *p* < 0.01), grey matter volume (r = −0.38; *p* < 0.01), and white matter lesion volume (r = 0.39; *p* < 0.01) within MS [53]. Higher sGFAP levels have been associated with greater lesion number (*p* < 0.01) in MS [40].

### 4.2. Expanded Disability Status Scale (EDSS)

The EDSS captures the global neurological burden of MS, and is applied for measuring disability and its progression over time in people with MS. Both sNfL and sGFAP have been associated with disease worsening based on EDSS scores [54,55]. Indeed, higher sNfL and sGFAP levels have been associated with increased disability accumulation (i.e., higher EDSS (B = 1.105 and *p* < 0.001; r = 0.36 and *p* < 0.001) [37,50,56,57]. One recent meta-analysis reported that those with higher baseline values of sNfL had a faster time for reaching an EDSS milestone of 4.0 (HR = 2.36, 95% CI = 1.32–4.21, and *p* < 0.005), nearly double that of those with “normal” concentrations of sNfL [36]. Conversely, higher levels of sGFAP have not been associated with a higher risk of progression (HR: 1.12, *p* = 0.052) [58].

### 4.3. Optical Coherence Tomography (OCT)

There has been some interest in sNfL and the integrity of cells in the retina based on optical coherence tomography (OCT). sNfL has been associated with lower retinal nerve fiber layer (RNFL) thickness (−3.03 ± 1.50 μm; *p* < 0.05) and a faster decline in thickness over time (45% faster; −0.74 vs. −0.51 μm/y; *p* < 0.05) [59]. There is limited and conflicting evidence on OCT and sGFAP in MS [60,61,62].

### 4.4. Cognitive Function

sNfL levels have been associated with cognitive functioning in MS. One recent meta-analysis investigated molecular biomarkers associated with cognitive function in MS and included data from 13 studies, of which 11 included NfL (CSF = 5; serum = 6). Of the studies assessing NfL, higher levels of sNfL were associated with slower cognitive processing speed [63]. There is further evidence that increased sNfL levels predicted faster decline in general cognitive functioning in MS [64].

There is limited evidence of sGFAP as a correlate of cognitive dysfunction. sGFAP concentrations have been weakly associated with cognitive performance (*p* = 0.072) and are not associated with nor predictive of future changes in information processing speed (SDMT) (*p* = 0.183 in univariate analysis; *p* = 0.850 in multivariate analysis) [58,65].

## 5. DMTs and Body Fluid Biomarkers of Neuroaxonal Injury in MS

DMTs are the first-line approach for managing MS disease activity and progression based on reducing relapse, lesions, and disease progression [66]. There are more than 25 DMTs currently available for MS, and the DMTs are generally classified into platform (moderate-efficacy) and high-efficacy therapies with varying effectiveness. Platform therapies are generally lower in potency than higher-efficacy therapies and have been typically used as the initial treatment for RRMS. High-efficacy therapies are more effective in lowering relapse rates and progression. sNfL has gained popularity as a measure of DMT effectiveness based on reducing neuroaxonal damage. sNfL levels have been reduced significantly with both platform [22.7 (17.5–39.1) to 20.2 (13.7–28.9) ng/L (*p* < 0.005)] and high-efficacy [17.7 (11.8–25.6) to 12.4 (8.3–19.7) ng/L (*p* < 0.001)] DMTs compared with untreated controls [38]. High-efficacy DMTs yielded greater reductions in sNfL levels, better MRI outcomes, and a decreased rate of EDSS advancement in a 4-year follow-up [38,56].

The effect of DMTs on sGFAP is less conclusive. There have been two studies examining evidence for DMTs’ effects on sGFAP, and both report no significant reduction in sGFAP with treatment [67,68]. The seeming lack of effect of DMTs on sGFAP compared with sNfL might be based on the latent damage resulting in expression of the biomarker. DMT’s yield suppression of acute inflammatory activity driven by the immune system prevents the formation of new lesions or areas of damage and reduces sNfL. However, GFAP is a marker of chronic inflammation (i.e., chronic gliosis) and disease progression and may not be readily affected by DMTs.

## 6. Exercise Training and Body Fluid Biomarkers of Neuroaxonal Injury in MS

ET represents a lifestyle, health behavior commonly applied for management of symptoms and rehabilitation among people with MS. ET has been associated with relapse rate, lesion number and volume, disability progression, and CPS in MS, thereby suggesting its potentially disease-modifying properties [6,8]. ET has further been associated with beneficial effects on BDNF, NGF, and CNTF, indicating its potential for neuroprotection and repair in MS [9].

ET has been examined for its beneficial effects on sNfL and sGFAP in MS, and we provide a description of all nine clinical trials of ET and those two biomarkers in Table 1. Of note, the studies included three RCTs and two secondary analyses of RCTs, and the remaining four were pilot studies or a quasi-experimental study. Sample sizes ranged from 11 to 86 participants. Most studies primarily included participants with RRMS with low to moderate disability (EDSS 0-6), with fewer participants with SPMS or PPMS subtypes of MS. Six of the studies included control conditions where the participants received no intervention or continued regular care, or there was no control condition. The remaining three conditions included comparators that were informational sessions with a medical professional, home-based core stabilization exercises, and comparisons between different exercise intensities (i.e., moderate compared with HIIT). Intervention modality varied across studies, with four including aerobic cycling exercise interventions, four including resistance training programs, and one study including a Pilates intervention. The exercise program duration varied across studies (3–24 weeks), with the shortest intervention lasting 3 weeks and the longest 24 weeks. The exercise session frequency was within the MS exercise guideline of 2–3 times per week of aerobic and resistance training, respectively [69]. All nine of the studies included analysis examining blood-based (serum or plasma) NfL levels, whereas only three included GFAP alongside NfL. Baseline, follow-up data, and variables of interest are included in Table 1. Of note, studies utilized both the ELISA (*n* = 3) and SIMOA (*n* = 6) assay platforms to quantify either NfL or GFAP levels. Of the nine studies, a mixture of sample media, including plasma (*n* = 3) and serum (*n* = 6), were used in the quantification of NfL or GFAP. Reporting sample times (the time of day at which samples were taken) and processing information (e.g., the centrifugation process) were either not reported or lacked clarity in the description of the procedures. The sample storage temperature was reported as −80 °C in seven studies, and −70 °C and −20 °C in one study each [12,70,71,72,73,74,75,76,77].

We located two meta-analyses, including the previously mentioned articles that examined the effects of ET on sNfL and sGFAP in MS [11,78]. The first meta-analysis included seven RCTs and focused on ET's effects on sNfL. ET was associated with positive changes in sNfL concentrations compared with controls (SMD = −0.55; 95% CI: −1.00, −0.09), and the largest effects were observed for outdoor Pilates (SMD = −2.08; 95% CI: −2.99, −1.17) and home-based Pilates (SMD = −1.46; 95% CI: −2.28, −0.64) [11].

The second meta-analysis included eight RCTs of ET programs (seven of which were identical to the aforementioned meta-analysis) with aerobic training's effects on concentrations of sNfL and sGFAP as outcomes, and the eighth RCT examined the effects of high-intensity circuit resistance training on sNfL and Tau protein [78]. Overall, ET intensity was associated with improved sNfL levels compared across training conditions. The moderator analysis utilized a surface under the cumulative ranking curve (SUCRA), which indicated that moderate-intensity exercise yielded the largest effect (SUCRA = 92.2; credibility interval: 75.3–95.2) for lowering sNfL levels, followed by high-intensity (SUCRA = 54.0; credibility interval: 50.1–89.1), and then low-intensity (SUCRA = 3.6; credibility interval: 29.8–89.0) exercise. There were conflicting and nonsignificant results for ET's effects on sGFAP [78].

**Figure 2:**
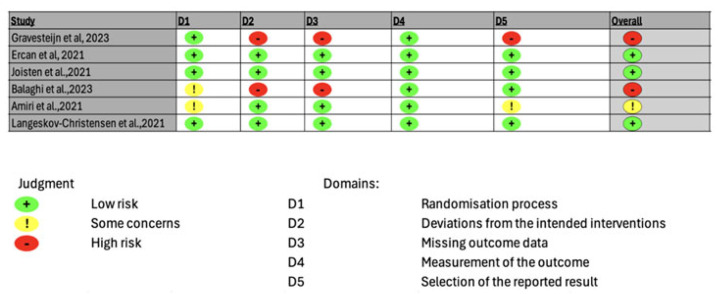
Risk of Bias Traffic Light Plot and Summary Graph for Randomized Controlled Trials of Exercise Training and Biomarkers of Neuroaxonal Injury in Multiple Sclerosis [12,70,71,73,74,76].

We conducted a formal risk-of-bias assessment that is included in Figure 2. The risk-of-bias was undertaken in only six of the nine studies, as these were RCTs; the three remaining studies were not included based on the study designs not fitting the RoB2 tool [79]. Of the studies assessed, three had a low risk of bias, one displayed some concerns, and the final two studies had a high risk of bias. Three of the studies by Ercan et al., Joisten et al., and Langeskov et al. received a low risk-of-bias determination [12,71,76]. The study that received some concern determination was conducted by Amiri et al., and the determination was driven by inadequately addressing issues surrounding concealment of allocation during the randomization process, and a bias in the selection of the reported results [74]. The studies by Gravesteijn et al. and Balaghi et al. received a high risk-of-bias assessment based on inadequately including relevant information across domains 2 (deviations from intended interventions), 3 (missing outcome data), and 5 (selection of the reported result) of the RoB2 tool, preventing a determination [70,73].

We further identified several methodological limitations across studies, including issues related to randomization, blinding, power analyses and appropriate sample sizes, and controlling for DMT status or relapse. Randomization reporting was inconsistent across studies, where most studies employed randomization but did not describe the procedures used. There was limited and inconsistent blinding across studies for outcome assessors or participants. Only four of the nine studies conducted an a priori power analysis, and in some cases, power analyses were described in the original trial protocols, but not the secondary analyses. Control of DMT status and relapse activity was addressed inconsistently across ET trials. Several of the studies required stable DMT and/or absence of relapse within a pre-specified period or reporting of relapse during follow-up periods. Others only reported DMT status without controlling for relapse activity, and others did not report either.

## 7. Discussion

This narrative review summarized the current understanding of two body-fluid biomarkers of interest for understanding ET's effects on disease activity in MS. sNfL and sGFAP are leading candidate biomarkers for clinically tracking disease activity and informing disease prognosis based on underlying neuroaxonal damage in MS. We summarized the evidence indicating that concentrations of both biomarkers are higher in MS than in controls and vary by different disease types and activity (i.e., with sGFAP being higher in progressive forms of MS (i.e., SPMS and PPMS) and sNFL being higher during a relapse). We further reported on demographic/morphological/clinical factors (e.g., age, BMI, DMT status, biological sex, and ethnicity) that moderate levels of those biomarkers, and that may serve as covariates and/or secondary variables in clinical research involving ET in MS. The two biomarkers co-express with imaging biomarkers and cognitive functions, as other markers of MS disease status, and this may provide a more holistic perspective of disease monitoring within ET clinical trials. We, lastly, explored the effect of ET on sNfL and sGFAP, and there are promising outcomes of RCTs, but much remains unknown, thereby supporting (a) trial design considerations and (b) application of a discovery model combined with an experimental medicine framework for identifying pathways between ET and body fluid biomarkers of neuroaxonal injury in MS [21,22].

### 7.1. Trial Design Considerations

Our review of the literature regarding body fluid biomarkers highlights the potential utility of sNfL and sGFAP as measures for quantifying disease activity and responsiveness in ET interventions involving people with MS. Of note, this narrative review identified several areas for informing future trials of ET and body fluid biomarkers in MS that we believe will enhance the quality and reproducibility of research going forward. One area involves consideration of factors that may influence or confound interpretation of body fluid biomarker levels, notably age and BMI. sNfL and sGFAP levels increase linearly with age, until 60 years, after which there is an exponential increase in circulating levels [80]. Those body fluid biomarkers further change based on BMI [81]. We recommend that biomarker levels be analyzed using either z-scores or percentiles that control for the effects of age and BMI to minimize those factors as extraneous influences on variation of biomarkers. Importantly, normative databases for comparison using healthy controls are already established [43].

There is a further need for better reporting of the procedures for acquiring, processing, and reporting body fluid biomarkers of neuroaxonal injury with ET in MS. This is necessary for the scientific rigor and reproducibility of ET trials. To that end, future trials should include specific timing of blood sampling, the methods used to process (i.e., centrifugation metrics) and store (temperatures) samples, and an assay identification number. This should be accompanied by documenting and controlling the presence of recent inflammatory activity or relapses, comorbidities, and the current type of DMTs.

Our examination of the nine ET trials provided initial evidence for a positive biomarker signal, notable in NfL. However, there was substantial heterogeneity between ET interventions, and only three of nine studies examined GFAP. This clearly supports the need for more standardized ET interventions and a focus on GFAP along with NfL, as this might identify a specific or general effect of ET on body fluid biomarkers of neuroaxonal injury. The lack of an effect of ET on GFAP may be reflective of the design of the studies, as both included samples largely consisting of participants with RRMS and lower numbers of progressive forms of MS (i.e., SPMS and PPMS). This may be an important caveat as GFAP is higher in more progressive forms of MS, and appropriate inclusion of people with SPMS and PPMS with confirmed higher disease burden (i.e., progressive atrophy and higher lesion burden) may help identify if ET influences GFAP levels.

To date, concrete recommendations for the ET stimulus that may produce a positive biomarker signal in blood-based biomarkers, like sNfL and sGFAP, are not available in the literature. The initial findings from the nine RCTs, and the other positive associations between ET and relapse rate, lesion number and volume, disability progression, and CPS, suggest that ET impacts established clinical outcomes within MS and, further, may influence sNfL levels [6,8,12,70,71,72,73,74,75,76,77]. This study presents some recommendations for future trials. Researchers might consider different exercise modalities (i.e., aerobic, resistance, and mixed), intensities (i.e., moderate versus high intensity), and durations (i.e., short, moderate, or long durations) within both acute and chronic paradigms for identifying ET programs that maximize benefits in body fluid biomarkers of neuroaxonal injury and clinical endpoints. The inclusion of clinical endpoints is critical for providing context alongside the changes in body fluid biomarkers of neuroaxonal injury with ET in MS.

### 7.2. Discovery Model and Experimental Medicine Model for Pathways of ET's Effect on Biomarkers

One unresolved issue is the pathway between ET and changes in body fluid biomarkers of neuroaxonal injury in MS. Indeed, researchers should identify the possible biological signal from muscle contraction and/or substrate utilization (metabolism) during ET that would facilitate changes and then adaptations in body fluid biomarkers of neuroaxonal injury. The primary candidates for consideration include the irisin and kynurenine pathways. Irisin is an exercise-induced myokine whose secretion is dependent on a signaling cascade starting with muscular contraction. Muscle contraction stimulates the receptor proliferator-activated receptor gamma coactivator 1-alpha (PGC-1α), which, in turn, induces an increased expression of fibronectin type III domain-containing protein 5 (FNDC5). FNDC5 is then cleaved, producing irisin, which is then released into circulation. Irisin, once in circulation, exerts a variety of effects across multiple body systems, including crossing the blood–brain barrier (BBB) into the CNS and promoting the expression of neuroprotective molecules like brain-derived neurotrophic factor (BDNF) [13,14,82]. This increase in BDNF might play a central role in regulation of neuroaxonal injury.

The kynurenine pathway is another possible explanation for ET's effects on body fluid biomarkers of neuroaxonal injury. That pathway involves tryptophan metabolism resulting in NAD+ production; however, other metabolites that are produced in this signaling cascade can exert varying effects within the CNS, encouraging a neuroinflammatory or anti-inflammatory environment [19,83]. Exercise and muscular contraction may cause a shift in the kynurenine pathway toward a more anti-inflammatory profile within the CNS, potentially resulting in neuroprotection [18]. Collectively, research exploring exercise and its potential effect on the irisin and kynurenine pathways alongside biomarkers of disease activity may offer a more complete understanding of how ET may influence the CNS and, in turn, clinical endpoints.

The discovery model offers a highly focused approach for identifying and exploring targets across multiple physiological systems for immediate (acute) and chronic (long-term) changes with exercise [21]. Using this model, researchers apply acute or single bouts of exercise as a stimulus for perturbation of a system or pathway in either a focal (specific) or exploratory (non-specific) manner. The acute bouts (i.e., stimulus) can vary in mode, intensity, and/or duration of exercise for identifying and exploring targets across multiple physiological systems (i.e., systems perturbed by a stimulus). This then informs the design of a chronic ET program, wherein researchers examine if repeated acute bouts of the identified stimulus over time result in sustained or long-term adaptations (i.e., general adaptation syndrome) [84]. Within the context of sNfL, as an example, one could examine changes in body fluid biomarkers, or precursor pathways (e.g., the kynurenine pathway as part of tryptophan metabolism), with acute bouts of exercise, as this could inform a RCT of a highly-informed chronic ET (i.e., repeated acute bouts over time) program when examining effects on body fluid biomarkers and precursors for established disease modification in MS [21].

The experimental medicine model is informed by the discovery model approach and outlines a four-step process for establishing mechanisms for ET outcomes [22]. Step 1 involves identifying CNS targets of interest and hypothesis generation, as done in this paper. Step 2 focuses on validating the CNS targets as correlates of outcomes of interest, for example, cognition or MRI in the context of ET. Step 3 focuses on examining the effects of ET on the chosen CNS targets. Step 4 integrates the information of the previous three steps and examines the effect of exercise on pertinent outcomes of interest with the CNS target(s) serving as a mediator(s). This framework, combined with the discovery model, offers unique opportunities for examining the effects of ET on clinical endpoints, such as mobility, MRI outcomes, cognitive function, and OCT, while using neurofilaments or other biomarkers of pathway metabolism as potential mediators.

We propose a path forward in the study of ET and blood-based biomarkers of neuroaxonal injury in MS. This is based on the discovery model and experimental medicine frameworks. The first step is the adoption of the discovery model and identifying the parameters of the ET stimulus via acute bouts of exercise that vary in modality, intensity, and/or duration for changes in pathway molecules, but not NfL or GFAP, as these are unlikely candidate markers for change with single bouts of exercise. Once the ideal stimulus parameters are identified, the next step is to utilize the experimental medicine framework and apply the ideal stimulus in a series of smaller RCTs of ET for examining changes in pathway molecules, plus neurofilaments like NfL and GFAP. The series of RCTs of ET should include hypothesis-driven inclusion of specific clinical metrics appropriate for addressing specific questions of a given study. Common clinical endpoints used in the population with MS, such as physical and cognitive function measures, as well as MRI and/or OCT as measures of CNS structure, would be appropriate. The samples for such research should be selected carefully based on the demographic, morphologic, and clinical factors that influence neurofilament levels, and this should include a rationale for age and BMI, as well as the type, duration, and course of MS, with sample selection parameters.

### 7.3. Limitations

There are limitations to the current review. Our review and selection of studies was not systematic in nature, but was guided by features of a narrative review for creating an argument for expanding and improving the limited pool of studies examining ET and blood-based biomarkers of neuroaxonal injury in MS; we did include all available research involving ET and neurofilaments in MS based on the literature search and meta-analyses [11,78]. The scope of this narrative review involved only ET, and we did not include other health behaviors based on an absence of systematic, published evidence. Another limitation is the non-specific nature of sNfL and sGFAP regarding MS disease pathology, as those markers are relevant in other neurodegenerative conditions (i.e., Alzheimer’s and other dementias, amyotrophic lateral sclerosis, Huntington’s disease and traumatic brain injury) [85,86]. One final limitation of this review is the limited pool of literature for sGFAP, compared with sNfL, as a marker of disease activity in people with MS.

## 8. Conclusions

The current paper provides a framework for better understanding ET and blood-based biomarkers on neuroaxonal injury and damage (i.e., sNfL and sGFAP) in MS. The results from early clinical trials, summarized in Table 1 and meta-analyses are generally promising [11,78] and support the future application of the discovery model and experimental medicine framework within this emerging line of research. Such a line of research would extend our understanding of the benefits of ET in MS and further position ET as a health behavior that could be implemented alongside DMTs within the clinical armamentarium of MS management.

## Figures and Tables

**Table 1:** (**a**) Summary of interventions and biomarker findings from the 8 studies examining exercise training's effects on sNfL and sGFAP. (**b**) Summary of biomarker outcomes.

(a)									
Paper	Study Design	Sample Size	MS Subtype	EDSS	Exercise Type	Exercise Frequency	Program Duration	Exercise Intensity	Program Type
Gravesteijn et al. [70]	Secondary Analysis of an RCT	*n* = 55 Aerobic training (*n* = 30) Control group (n = 25)	Aerobic: RRMS = 17 PMS * = 8 Unknown = 5 Control: RRMS =16 PMS * = 8 Unknown = 1	Aerobic: 2.5 (2.0,3.0) Control: 3.0 (2.0,3.5) Median (IQR)	Aerobic (cycling)	3×/week	16 weeks	30 min in total; 6 intervals (5 min of cycling); 3 min at 40% peak power; 1 min at 60% PP; 1 min at 80% PP	Mixed
Ercan et al. [71]	RCT	*n* = 38 Aerobic training (*n* = 19) Control (*n* =19)	Aerobic: RRMS = 19 Control: RRMS = 19	Aerobic: 1.90 ± 1.11 Control: 2.05 ± 0.98	Aerobic (cycling)	3×/week	8 weeks	40 min in total; 5 min warm-up at 20% of VO2 max.; 30 min at 60–70% of VO2 max.; 5 min cooldown	Mixed
Joisten et al. [12]	Secondary analysis of an RCT	*n* = 69 HIIT (*n* = 35) MCT (*n* = 34)	HIIT: RRMS = 21 SPMS = 14 MCT: RRMS = 21 SPMS = 13	HIIT: 4.44 ± 1.06 MCT: 4.59 ± 1.08	Aerobic (cycling)	3×/week	3 weeks	3 min warm-up; 5 HIIT intervals; 1.5 min at 95–100% of HRmax; 2 min unloaded between intervals; 3 min cool down	Supervised
Mulero et al. [72]	Longitudinal study	*n* = 11	RRMS = 11	0 (0–2) Median (IQR)	Resistance	3×/week	6 weeks	7 resistance exercises; 5 min warm-up; 3 sets × 8–10 repetitions at 70–80% of 1RM;	Supervised
Balaghi et al. [73]	RCT	*n* = 44 Outdoor Pilates (*n* = 15) Home-based Pilates (*n* = 15) Control (*n* = 14)	RRMS = 44	2–5 Range	Pilates	3×/week	8 weeks	60 min total per session; 10 min warm-up (7 movements); 40–45 min of 14 movements; 10 min cooldown	Home-based
Amiri et al. [74]	Quasi-experimental	*n* = 24 Resistance (*n* = 12) Control (*n* =12)	N/A	2–5 Range	Resistance	3×/week	8 weeks	45–60 min total per session; 10 min warm-up; 10 resistance exercises; 2 sets of 10–12 repetitions at 45–55% 1RM; 10 min of cool down	Supervised
Maroto-Izquierdo et al. [75]	Longitudinal pilot study	*n* = 11	RRMS = 11	0.5 ± 0.8	Resistance	3×/week	6 weeks	7 Resistance exercises; 5 min warm-up; 3 sets × 8–10 repetitions at 70–80% of 1RM	Supervised
Langeskov-Christensen et al. [76]	RCT	*n* = 86 PAE (*n* =43) Waitlist (*n* = 43)	PAE: RRMS = 41 PPMS = 2 SPMS = 0 Waitlist: RRMS = 34 SPMS = 5 PPMS = 4,	PAE 2.7 ± 1.4 Waitlist 2.8 ± 1.6	Aerobic (cycling)	2×/week	24 weeks	30–60 min over the course of the program; 5 min warm-up; cycling intensity was progressive from 65% to >95% of HRmax	Mixed
Gravesteijn et al. [77]	Extended baseline study	*n* = 30	SPMS and PPMS *n* = 30	4.5 (4.0–5.5) Median (IQR)	Resistance	3×/week	16 weeks	60 min session: 5 min warm-up; 6 exercises: 3–4 sets × 10–12 repetitions at 60–80% of 1RM; 10 min cooldown	Mixed
**(b)**									
**Paper**	**Biomarker**	**Assay Platform**	**Sample Medium**	**Sample Time**	**Sample Processing**		**Storage Temperature**	**Baseline**	**Follow-Up**
Gravesteijn et al. [70]	NfL GFAP	SIMOA	Serum, pg/mL	9.00–17.00	Centrifuged at 2000× *g* 10 min		−80 °C	sNfL: 8.8(5.3;14.2) sGFAP: 97.2(72.8;137) Median (IQR)	sNfL: 7.5(5.9;12.5) sGFAP: 93.3(68.8;140) Median (IQR)
Ercan et al. [71]	NfL sGFAP	ELISA	Serum, ng/mL	Morning (unspecified hours)	Centrifuged at 4000× *g* 5 min		−80 °C	sNfL: 7.26 ± 6.22 sGFAP: 3.058 ± 2.505	sNfL: 5.476 ± 4.245 **# sGFAP: 2.786 ± 2.841 *
Joisten et al. [12]	NfL	SIMOA	Plasma, pg/mL	8.00–9.00	Centrifuged at 3500 rpm × 10 min		−80 °C	T0: 11.869 ± 6.669	T1:10.53 ± 5.89 **## T2:11.00 ± 7.00 **## T3:12.41 ± 7.81
Mulero et al. [72]	NfL	SIMOA	Plasma, pg/mL	NR	NR		−80 °C	6.61(3.64) Median (IQR)	(1-wk post): 4.44(1.91) ** (4-wk post): 4.38(2.12) * Median (IQR)
Balaghi et al. [73]	NfL	ELISA	Serum, ng/mL	48 h before training	NR		−70 °C	OPT:21.35 ± 2.74 HPT: 22.01 ± 3.01	OPT:15.98 ± 2.26 # HPT: 18.01 ± 2.28 #
Amiri et al. [74]	NfL	ELISA	Serum, ng/mL	24 h before first session, and 48 h after last session	NR		−20 °C	65.92 ± 7.94	62.36 ± 5.75
Maroto-Izquierdo et al. [75]	NfL	SIMOA	Serum, pg/mL	NR	NR		−80 °C	T1: 5.3 ± 1.8	T2(1-week post): 5.9 ± 2.0 ## T3(4-week post): 4.2 ± 1.7 ##
Langeskov-Christensen et al. [76]	NfL	SIMOA	Serum, ng/L	9–11 am	NR		−80 °C	T0: 8.2 (6.7;11.4) Median (IQR)	T(24): 8.6(6.8;12.8) T(48): 8.3(6.8;11.2) Median (IQR)
Gravesteijn et al. [77]	NfL GFAP	SIMOA	Plasma pg/mL	NR	Centrifuged at 10,000× *g* 10 min		−80 °C	T0: NfL: 10.696 ± 5.235; GFAP: 99.9 ± 44.87	T1: NfL: 10.88 ± 4.876 GFAP: 92.171 ± 40.96 T2: NfL: 10.92 ± 5.174 GFAP: 94.124 (46.52) T3: NfL: 10.329 ± 4.048 GFAP: 97.092 ± 51.804

Abbreviations: Relapse-remitting multiple sclerosis (RRMS), secondary progressive multiple sclerosis (SPMS), primary progressive multiple sclerosis (PPMS), peak power (PP), maximal oxygen consumption (VO2max), 1-repetition max. (1-RM), maximum heart rate (HRmax), high-intensity interval training (HIIT), moderate-intensity continuous training (MCT), and progressive aerobic exercise (PAE). (+) indicates a significant reduction in biomarker levels after exercise training, and (-) indicates no significant reduction in biomarker levels after exercise training. * PMS was used if the sample included participants with progressive MS but did not report specific disease subtypes. Neurofilament light chain (NfL), glial fibrillary acidic protein (GFAP), enzyme-linked immunosorbent assay (ELISA), single-molecule enzyme-linked immunosorbent assay (SIMOA), and not reported (NR). ** Note: Data from baseline and follow-up results are reported as means ± st. deviation. Any other form reported was specified within the cell. **/*—significant within group with *p* < 0.05, **—significant within group with *p* < 0.01, #—significant between groups with *p* < 0.05, and ##—significant between groups with *p* < 0.01.

## Data Availability

No new data were created or analyzed in this study. Data sharing is not applicable to this article.

## Supplementary Materials

The following supporting information can be downloaded at https://www.mdpi.com/article/10.3390/ijerph23030380/s1: Supplemental Document S1.

## Abbreviations

The following abbreviations were used in this manuscript:

BMI	Body Mass Index
BDNF	Brain-Derived Neurotrophic Factor
CNS	Central Nervous System
CNTF	Ciliary Neurotrophic Factor
CIS	Clinically Isolated Syndrome
CPS	Cognitive Processing Speed
DMT	Disease-Modifying Therapy
ELISA	Enzyme-Linked Immunosorbent Assay
ET	Exercise Training
EDSS	Expanded Disability Severity Scale
FNDC5	Fibronectin Type III Domain-Containing Protein-5
HIIT	High-Intensity Interval Training
HRmax	Maximal Heart Rate
MCT	Moderate Continuous Training
MS	Multiple Sclerosis
NAD^+^	Nicotinamide Adenine Dinucleotide
NGF	Nerve Growth Factor
PGC-1α	Proliferator-Activated Receptor Gamma Coactivator 1-Alpha
PDDS	Patient Disability Status Scale
PMS	Progressive Multiple Sclerosis
PPMS	Primary Progressive Multiple Sclerosis
QOL	Quality of Life
RRMS	Relapse-Remitting Multiple Sclerosis
RNFL	Retinal Nerve Fiber Layer
sGFAP	Serum Glial Fibrillary Acidic Protein
SIMOA	Single-Molecule Enzyme-Linked Immunosorbent Assay
sNfL	Serum Neurofilament Light Chain
SMD	Standardized Mean Difference
SDMT	Symbol Digit Modalities Test
SPMS	Secondary Progressive Multiple Sclerosis
VO2max	Maximal Oxygen Consumption
