# Supplementary material for: Exercise Training and Biomarkers of Neuroaxonal Injury in Multiple Sclerosis: Narrative Review
Source: Int J Environ Res Public Health. 2026 Mar 17;23(3):380. doi: 10.3390/ijerph23030380 (PMC13026328; doi:10.3390/ijerph23030380)
Supplement: Supplementary file 1 [file ijerph-23-00380-s001.zip › ijerph-4140514-supplementary.pdf]

PubMed String

("Multiple Sclerosis"[Mesh] OR "multiple sclerosis" OR MS) AND ("Exercise"[Mesh] OR "Exercise Therapy"[Mesh] OR "Physical Exertion"[Mesh] OR "Motor Activity"[Mesh] OR exercise OR "physical activity" OR training OR aerobic OR endurance OR rehabilitation) AND ("Neurofilament Proteins"[Mesh] OR neurofilament* OR "neurofilament light" OR NfL OR GFAP OR "glial fibrillary acidic protein")

EBSCOhost Search String

((MH "Exercise+") OR (MH "Exercise Therapy+") OR TX (exercise* OR "physical activity" OR aerobic OR training)) AND

((MH "Multiple Sclerosis+")) AND

((MH "Neurofilament Proteins+") OR TX ("neurofilament light chain" OR "neurofilament light" OR NfL) OR TX (GFAP OR "glial fibrillary acidic protein")) AND

(TX (serum OR plasma OR blood))

EMBASE Search String

('exercise'/exp OR 'exercise therapy'/exp OR (exercise* OR "physical activity" OR aerobic OR training):ti,ab,kw) AND

('multiple sclerosis'/exp) AND

('neurofilament protein'/exp OR "neurofilament light chain":ti,ab,kw OR "neurofilament light":ti,ab,kw OR nfl:ti,ab,kw OR gfap:ti,ab,kw OR "glial fibrillary acidic protein":ti,ab,kw) AND

(serum:ti,ab,kw OR plasma:ti,ab,kw OR blood:ti,ab,kw)
